# Effects of Circuit Weight-Interval Training on Physical Fitness, Cardiac Autonomic Control, and Quality of Life in Sedentary Workers

**DOI:** 10.3390/ijerph18094606

**Published:** 2021-04-27

**Authors:** Silvio A. Oliveira-Junior, Daniel Boullosa, Maria L. M. Mendonça, Larissa F. C. Vieira, Wania W. Mattos, Bruna O. C. Amaral, Dayanne S. Lima-Borges, Filipe A. Reis, Marcelo D. M. Cezar, Luiz C. M. Vanderlei, Paula F. Martinez

**Affiliations:** 1 Integrated Institute of Health, Federal University of Mato Grosso do Sul—UFMS, Campo Grande 79070-900, MS, Brazil; daniel.boullosa@gmail.com (D.B.); marialuamarques@hotmail.com (M.L.M.M.); larissafcv@gmail.com (L.F.C.V.); wania.wein@gmail.com (W.W.M.); brunaamaral02@hotmail.com (B.O.C.A.); limaborgesds@gmail.com (D.S.L.-B.); paula.martinez@ufms.br (P.F.M.); 2 Department of Physical Therapy, Anhanguera University—UNIDERP, Campo Grande 79003-010, MS, Brazil; fiabdalla@gmail.com; 3 Department of Physical Education, Itapeva Social and Agrarian Sciences College—FAIT, Itapeva 18412-000, SP, Brazil; marccezar@hotmail.com; 4 Department of Physical Therapy, Sao Paulo State University—UNESP, Presidente Prudente 19060-900, SP, Brazil; lcm.vanderlei@unesp.br

**Keywords:** sedentary behavior, autonomic modulation, exercise, quality of life, physical fitness

## Abstract

Sedentary behaviors, those that involve sitting and low levels of energy expenditure, have been associated with several adverse cardiometabolic effects. This study evaluated the chronic effects of a combined circuit weight interval training (CWIT) on physical fitness, quality of life, and heart rate variability (HRV), and compared the effects of CWIT-induced autonomic adaptations on different postures in adult sedentary workers. Twenty-seven sedentary workers (age 36.9 ± 9.2 years old, 13 men and 14 women) were divided into two groups: control, who continued their sedentary behavior, and experimental, who were submitted to a CWIT for 12 weeks, completing two ~40 min sessions per week. Monitoring of 8th, 16th, and 24th sessions revealed a moderate training load during sessions. Participants exhibited an improved aerobic capacity (VO_2_max, 34.03 ± 5.36 vs. 36.45 ± 6.05 mL/kg/min, *p* < 0.05) and flexibility (22.6 ± 11.4 vs. 25.3 ± 10.1 cm, *p* < 0.05) after the training period. In addition, they showed greater quality of life scores. However, the CWIT did not change body composition. Interestingly, more HRV parameters were improved in the seated position. The CWIT used in the current study was associated with improvements in several fitness and quality of life parameters, as well as in cardiac autonomic control of HR in adult sedentary workers. Examination of different body positions when evaluating changes in HRV appears to be a relevant aspect to be considered in further studies. Future randomized controlled trials (RCTs) with larger samples of both sexes should confirm these promising results.

## 1. Introduction

Sedentary behavior refers to any activity in a reclining, seated, or lying position requiring very low energy expenditure, and has been associated with several adverse metabolic effects as obesity, hyperglycemia, hyperlipidemia, and high cardiovascular risk [1,2]. Cardiometabolic diseases resulting from a sedentary lifestyle have a slow progress and take a long period to be detectable [3]. This picture is further complicated when referred to sedentary workers with limited time for leisure activities who spend most time of the day seated at work [4]. Previously, the use of different training programs at the workplace to counteract the negative impact of sedentary behavior associated to working tasks has been suggested [5,6,7,8]. These previous studies included the use of different exercise modalities as endurance training [5], strength and resistance training protocols [7], and combined exercise interventions [6,8]. However, there is no consensus on what training strategies may be more effective, from a dose-response perspective, especially when considering the inherent limitations of working schedules and reduced facilities for physical training at workplaces.

Heart rate variability (HRV) is a sensitive tool to monitor cardiac autonomic adaptations under different conditions [9]. The study of HRV is based on the measurement of heartbeat fluctuations over time and mainly reflects the modulation of the parasympathetic nervous system [9,10,11]. Reduced HRV is associated with increased all-cause mortality, and risk of heart failure, myocardial infarction, hypertension, and mental health problems [10,11], as well as impaired quality of life [12]. Moreover, HRV reductions have been correlated with excessive fat accumulation [13] and stress in response to work demands [14,15,16]. The positive impact of aerobic exercise and more specifically, high intensity interval exercise on several HRV parameters has been previously documented [17,18,19,20]. Meanwhile, although less documented, it seems that resistance training has also showed positive autonomic changes apart from the expected neuromuscular adaptations [21,22].

The enhancement of vagal modulations via different aerobic exercises in sedentary subjects have been associated to positive outcomes as increased physical fitness, health related parameters, quality of life, and stress resilience [11,23,24,25]. Interestingly, the influence of these exercise-derived autonomic adaptations on different postures has not been addressed yet. Previous studies, mainly in sport settings, have revealed differences regarding HRV outcomes between postures [26,27,28]. This aspect is relevant since the most evaluated posture is the supine posture, a condition that maximizes vagal modulations. In contrast, there are few studies evaluating the effects of different exercise modes on HRV in the seated posture [29,30], which is the posture in which white-collar employees spent more time at work. Previously, Tonello et al. [11] revealed some correlations between measures of HRV and cardiorespiratory fitness (CRF), which differed between the seated and the standing postures in sedentary female workers. More recently, Medeiros et al. [31] showed that the body position for HRV evaluations may influence the strength of the correlations with physical fitness and physical activity parameters. Therefore, the evaluation of exercise-derived autonomic adaptations in workers in different postures is a relevant topic that merits more attention.

Combined circuit weight-interval training (CWIT) is a multimodal exercise workout based on the combination of traditional circuit weight training and interval training and has been demonstrated to be more efficient for increasing physical fitness than other traditional exercise modalities [32]. Previously, a traditional circuit weight training increased muscle strength, but with only moderate improvements in terms of aerobic capacity [33]. On the other hand, interval training has been shown to further increase aerobic capacity compared to traditional, long-duration, submaximal endurance training [34]. Therefore, adding interval training into a traditional circuit weight training may further enhance the benefits of a pure circuit weight training by placing increased demands upon the cardiovascular system [35]. Concurrent training has previously shown a significant improvement in vagal indices in middle-aged hypertensive women [36]. Moreover, CWIT intervention has been associated with higher blood lactate levels and increased post-exercise oxygen consumption, when compared to other traditional circuit training protocols [32]. Further, regular exercise has also been associated with several benefits on the health and quality of life of office workers [37]. However, to the best of our knowledge, the impact of CWIT on physical fitness, quality of life, and HRV parameters in sedentary workers has not been investigated yet. It may be expected that this new method could improve, more efficiently, both aerobic and anaerobic components of physical fitness, as well as HRV and quality of life. This information would be important to better design intervention strategies in workplaces aiming to improve health related outcomes of sedentary workers with limited time to exercise.

Thus, the main objective of the present study was to analyze the effects of CWIT on physical fitness, quality of life, and HRV in adult sedentary workers. A secondary objective was to evaluate and compare the effects of exercise induced autonomic adaptations on different postures. It was hypothesized that CWIT would improve quality of life and several facets of physical fitness and cardiac autonomic modulation in the different postures evaluated.

## 2. Materials and Methods

### 2.1. Participants

**Figure 1:**
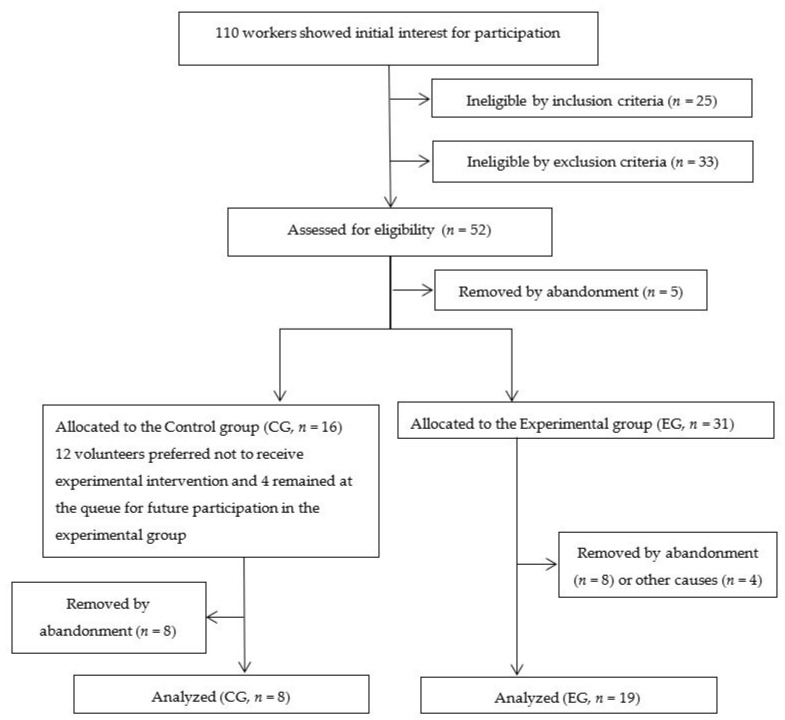
Study flowchart of participants.

One hundred and ten workers from a Brazilian higher education institution were invited to participate in this study. A total of 27 healthy sedentary workers (13 men and 14 women, 21–56 years-old) finally accepted the invitation (see Figure 1). Eligible volunteers had to be >18 years old, sedentary, have been working in their current job during at least 6 months prior to the start of the study, and to work in the seated position ≥4 h/day. In addition, to characterize a sedentary lifestyle, participants had to be engaged in less than 150 min of moderate-intensity physical activity per week [38] during, at least, three months. Exclusion criteria included being pregnant, having any kind of cardiorespiratory, metabolic, neuromuscular, or endocrine disease, being a smoker, or any clinical condition within 6 months prior to the start of the study. None of the participants was taking any medication that would affect cardiovascular responses. Any contraindication for exercising was assessed with the Physical Activity Readiness Questionnaire (PAR-Q) [39]. Sample size calculation was performed following a similar previous study in our institution [25], in which a dropout rate of 32% was observed. Thus, for a small effect size (0.25), an α = 0.05, and a power = 80% for this study design, a minimum of 10 participants was required. Throughout the present study, there was a dropout rate of 38.5% due to factors such as abandonment, musculoskeletal injuries unrelated to the study, and reported lack of time (see Figure 1).

All participants gave their informed consent for inclusion before participation. The study protocol was completed in accordance with the Declaration of Helsinki and was approved by the Institutional Research Ethics Committee (Protocol no. 37573914.2.0000.0021) and was registered in the Brazilian Clinical Trials Registry (ReBEC; Primary ID Number: RBR-5NJNQT). Participants were divided into control group (CG, *n* = 8) and experimental group (EG, *n* = 19). The present study is a semi-randomized controlled trial according to the Consort Statement [40]. Figure 1 illustrates the flowchart of the experimental research. Analytical procedures and interventions were performed in the Integrated School Clinic, situated in the Integrated Health Institute (INISA) of the Federal University of Mato Grosso do Sul in Campo Grande/MS (Brazil).

### 2.2. Outcome Measures

The exercise training intervention was linearly periodized following the basis of the circuit weight-training workout proposed by Skidmore et al. [32]. The protocol consisted of an initial warm-up in the cycle ergometer (Biotec 2100, Cefise^®^, Brazil) with a load corresponding to 1% of body mass; the intensity of warm-up was set at 60–70% of the age-predicted maximum heart rate (HRmax) [10,41]. Subsequently, the daily training protocol involved nine exercises arranged in three mini-circuit stations plus a cooling down exercise in the cycle ergometer (no load; 50–70 rpm per 5 min) and global stretching exercises (~9 min). Completion of each training protocol session took ~40 min and was scheduled in eight stages with three exercise stations, A, B, and C. The sequence of stages and their respective activities are presented in Table 1. The participants of the Experimental group performed two CWIT sessions per week over 12 weeks, with each session separated by 48–72 h thus totaling 24 exercise sessions.

Rating of perceived exertion (RPE) scores were obtained using the Borg’s 6–20 RPE scale [42,43]. The participants were asked to rate how hard they felt at the start of the session, and after each exercise station of the circuit weight-training workout. In order to evaluate the HR response during exercise training, a HR monitor (Polar Electro, model FT1, Espoo, Finland) was used to record the HR during all the sessions. Both RPE and HR measurements were obtained during pre-exercise, and immediately following completion of each of the three circuits (Station A, B, and C) according to Table 1. In sessions 8, 16, and 24, HR and RPE at pre-exercise and following completion of each of three mini-circuit stations (Stations A, B and C) were used to evaluate the training load of the exercise training protocols.

### 2.3. Physical Fitness Evaluations

Baseline measurements were obtained from each participant, including information about age, anthropometry, and health-related quality of life parameters. Anthropometrical measurements were performed in a room under thermoneutral ambient conditions of 22–24°C and 40–60% of relative humidity. Participants were instructed to wear minimum clothes for all anthropometrical assessments. Height was measured with a portable stadiometer (Líder, LD 1050, Araçatuba, SP, Brazil), with precision of 0.1 cm. Body mass was measured on a digital scale (Welmy R-110, 2010 São Paulo, Brazil), with a precision of 0.1 kg. Body mass index (BMI) was subsequently was calculated using the following formula: body mass (kg)/height (m)^2^. Hip, waist, middle arm, and middle leg circumferences were measured using an anthropometrical tape [38,44]. First, participants were in orthostatic position and a measuring tape (Sanny^®^, São Bernardo do Campo, SP, Brazil) with precision of 1.0 mm was used to obtain body circumferences. Hip circumference was measured following the Canadian Standardized Test of Fitness (CSTF) protocol, at the level of the symphysis pubis and the greatest gluteal protuberance [44]. To determine waist circumference, the tape was placed on the mid-point between the last floating rib and the top of the iliac crest in the mid-axillary line to measure the waist circumference, following the World Health Organization (WHO) protocol [38]. Middle arm circumference was measured with a tape positioned at the midpoint between the acromion and the olecranon. To analyze middle leg circumference, the tape was positioned at the maximum perimeter of the calf muscle of the right leg. The Medical Study 36-item Short-Form Health Survey (SF-36) was used to evaluate the health-related quality of life [45].

The Rockport Fitness Test was used as a measure of aerobic fitness in order to estimate the VO_2_max [46] after completion of 1 mile on a track. A heart rate monitor (Polar Electro, model FT1, Finland) was used to record the HR during the test. The VO_2_max estimation was obtained from the following formula: VO_2_max (mL/kg/min) = 132.853 − (0.0769 × body mass (pounds)) − (0.3877 × age (years)) + (6.315 × sex) − (3.2649 × T) − (0.1565 × Final HR); where: “sex”, female = 0; male = 1; T, total time of test (min); Final HR, heart rate obtained after test.

The 1-min curl-ups test, a test of muscular endurance, was performed according to previously described methods [47]. Maximal isometric handgrip strength was recorded with the dominant hand using a handheld handgrip dynamometer (Saehan^®^, Smedley-Type, Masan, Korea), following the guidelines of the American Society of Hand Therapists [48]. The strength values were obtained in kilograms, with a precision of 0.1 kg. Participants were instructed to sit in a straight-backed chair with feet placed flat on the floor, shoulder adducted and neutrally rotated, elbow flexed at 90°, and the forearm and wrist in neutral position. Handgrip strength was measured three times with 1 min of rest between attempts, and the maximal value obtained was considered for comparisons [48]. The back mobility and flexibility of leg muscles were evaluated with the modified sit-and-reach test (i.e., Wells and Dillon’s Bench) [49].

### 2.4. Heart Rate Variability Analyses

After a 48–72 h interval, participants were individually assessed between 7:00 and 11:00 a.m., under thermoneutral ambient conditions of 22–24°C and 40–60% of relative humidity, to evaluate the HRV at baseline and after the experimental protocol. Participants were instructed to avoid alcoholic and stimulant drinks such as coffee or tea, 24 h before.

The electrodes were placed on the participant’s chest at the sternal angle using an elastic strap, which was connected via Bluetooth to a HR monitor (V800, Polar Electro Oy, Espoo, Finland). The equipment has been validated to record beat-to-beat recordings for its use with HRV data analyses [50]. Participants were instructed to remain quiet, in silence, with spontaneous and free breathing and refrain from sniffing, sighing, or other abnormal breathing patterns [51], while resting in supine and sitting positions for 20 min in each position. The beat-to-beat HR recordings were transferred to a computer and filtered using a commercial software (Polar Flow^®^, Polar, Espoo, Finland). For HRV analyses, the HR beats obtained during the first 2 min interval were discarded and a 10 min recording of HR beats was selected after manual artefact and ectopic beats correction when needed. Only series with >95% of normal sinus beats were included in the study. Cleaned data were then transferred to a dedicated HRV analytical software package (Kubios 2.2, The Biomedical Signals Analysis Group, University of Kuopio, Kuopio, Finland) to obtain linear and non-linear measurements [52].

The HRV parameters selected for analysis were standard deviation of normal–normal R-R beats (SDNN), and root mean square of the successive differences (RMSSD) as time domain parameters; sample entropy (SampEn), and exponent of short-term fractal scaling (α1) as nonlinear analyses; and the spectral components of the low (LF, 0.04–0.15 Hz) and high frequency (HF, 0.015–0.4 Hz) bands, total power (TP), and the LF/HF ratio, as the frequency domain parameters. The spectrum resulting from the fast Fourier transforms (FFT) modeling was derived from all the data present in the recorded signal [9]. A custom HRV analysis software (Kubios 2.0, Biosignal Analysis and Medical Image Group, Department of Physics, University of Kuopio, Kuopio, Finland) was used for filtering and analyzing the R-R data.

### 2.5. Statistical Analysis

The Kolmogorov–Smirnov test was applied in order to verify data normality assumptions. The HRV parameters were converted to a natural logarithm scale (ln) to ensure normal data distribution when appropriate. Age values were analyzed using Student’s *t*-test. A two-way ANOVA of repeated measures was adopted to analyze parametric results with moments and group as factors. When significant differences were found (*p* < 0.05), post hoc Bonferroni’s comparisons were performed. Partial eta squared (*η_p_*^2^) was calculated to determine the effect size: Small (≥0.0099 and <0.0588), moderate (≥0.0588 and <0.1379), and large effects (≥0.1379), respectively [53]. The Mann–Whitney test was used to compare non-parametric results. On the other hand, the within-group analysis (between moments) was conducted using the Wilcoxon test. The effect size of the nonparametric data was based on Z-score values and converted to an estimated effect size (*r*): *r* = Z/√N, where Z is Z-score and N corresponds to the number of observations. The Goodman’s test for contrasts between and within multinomial populations was used to identify differences in distribution between categories. The level of significance was considered to be 5%.

## 3. Results

The control and experimental groups aged 38.7 ± 10.6 and 36.1 ± 8.7 years, respectively. Of note, while control group was composed by three women (37.5%) and five men (62.5%), the experimental group had 11 women (57.9%) and 8 men (42.1%; *p* > 0.05). Anthropometrical results are presented in Table 2. Exercise training intervention did not change body mass, BMI, or arm, leg, waist, and hip circumferences (*p* > 0.05).

The exercise training increased maximal isometric handgrip strength, sit and reach test performance, 1-min curl-ups test performance, and estimated VO_2_max after the experimental period, when compared to the initial moment. Physical fitness testing exhibited large effect sizes in response to CWIT (Table 3).

**Figure 2:**
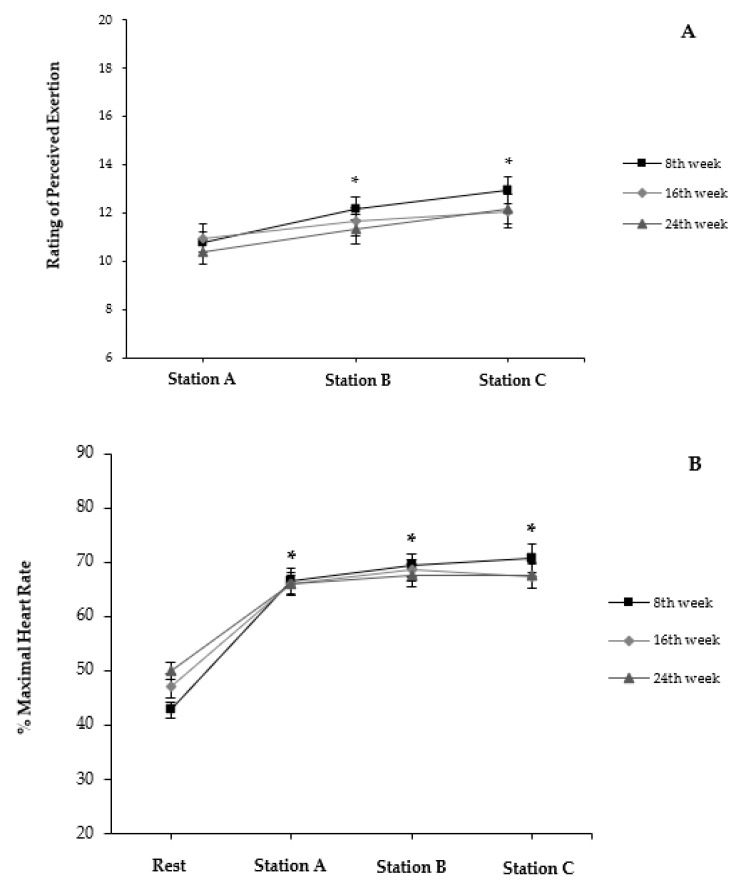
(**A**) Rating of perceived exertion and (**B**) % maximal heart rate at pre-exercise (Rest) and following completion of each exercise station (A, B, and C) during sessions 8, 16, and 24. Values expressed as mean and standard error; * *p* < 0.05 versus initial moment within session.

Ratings of perceived exertion (RPE) immediately following completion of each station during 8th, 16th, and 24th sessions are presented in Figure 2A. Within each session, values of RPE increased approximately by two scores after each station (*p* < 0.05). Profiles of responses were comparable among the sessions (*p* > 0.05). Heart rate (HR) for pre-exercise and immediately following the completion of each station during the 8th, 16th, and 24th sessions are presented as %HRmax in Figure 2B. No differences were identified for HR between sessions (*p* > 0.05). Values of HR at A, B, and C increased ~60.0% above pre-exercise levels (*p* < 0.05).

The CWIT significantly (*p* < 0.05) increased several facets of quality of life in the experimental group. Most changes exhibited large effect sizes in response to the CWIT intervention (Table 4).

Analyses of HRV changes in the supine position are presented in Table 5. The experimental group showed higher values of SDNN, LF, and TP after the exercise training intervention in comparison to the initial moment. These changes exhibited large effect sizes. Other HRV measures in time and frequency domains, as well as non-linear analyses were not changed by the exercise training intervention (*p* > 0.05).

Regarding the seated position, results of HRV are presented in Table 6. Interaction between group and moment was statistically significant (*p* < 0.05) for all time domain measures. The two measures were increased after the exercise training intervention when compared to the initial moment in the experimental group. Regarding the frequency domain measures, the experimental group presented a greater LF and TP after the training intervention when compared to the initial evaluation. All significant changes exhibited large effect sizes. Regarding the non-linear results, only detrended fluctuation analysis (DFA) Alfa-1 was increased between moments, in despite of the studied group (*p* = 0.040).

Based on HRV in sitting position, in terms of Δ% between moments of evaluation, the CWIT intervention promoted a significant increase only in HF (control, −4.84 ± 4.93; experimental, 6.57 ± 15.13; *p* = 0.029) and total power (control, −2.0 ± 6.8%; experimental, 7.4 ± 8.8%; *p* = 0.013).

Regarding relative variation between positions, RMSSD were lower in final in comparison to the initial moment within the control group. Other HRV parameters were not significantly changed following this analysis (see Table 7).

## 4. Discussion

According to initial hypothesis, the CWIT program improved physical fitness, quality of life, and HRV parameters in sedentary adult workers, with only 2 40-min sessions per week, over 12 weeks. Thus, the current intervention increased handgrip strength, muscle flexibility and endurance, as well as aerobic fitness (see Table 3). Furthermore, CWIT also resulted in greater scores of several facets of quality of life (see Table 4), and several HRV parameters in the supine (see Table 5) and seated (see Table 6) position. Of note, the HRV parameters were mostly increased in the seated position.

Exercise variables, such as frequency, intensity, and duration, have been previously manipulated in order to create an overload and to achieve a subsequent training effect [54]. In this context, HR is often used as a parameter to set exercise intensity due to the expected linear relationship between HR, VO_2_, and exercise intensity [54]. During the CWIT protocol, participants reached 85–90% of HRmax values during all stations, according to measures obtained after each session. Consequently, it is plausible that the volunteers had been working at an appropriate intensity throughout the training sessions, thereby stimulating cardiovascular adaptation. In this context, caution should be taken when using HR in an attempt to assess the intensity of resistance exercise alone [32], as CWIT is a combined exercise training protocol. According to Gotshalk et al. [55], CWIT produced HR values within the recommended range for developing cardiovascular fitness; however, the VO_2_ may not follow the same response in other exercises [56]. Therefore, future studies should consider both VO_2_ and blood lactate values in order to better describe the CWIT acute effects to better understand the chronic adaptations.

On the other hand, the RPE scale has been widely used as a method for estimating exercise intensity and evaluating exercise tolerance [42]. Although RPE has been used to monitor exercise intensity in response to other resistance training protocols [57,58], RPE have been only reported in a previous similar research using circuit training protocols [32]. When interpreting the RPE results from the current study, it should be noted that participants were asked to provide RPEs upon completion of each station (A, B, C), which also included the cycling exercise following each exercise station. It is noteworthy that the profile of the HR and RPE curves are relatively similar across the 8th, 16th, and 24th sessions with gradual increases observed at each exercise time point, therefore suggesting RPE could be an appropriate method for characterizing exercise intensity during CWIT protocols.

Several changes in the work environment have contributed to an increase in sedentary behavior accompanied by a decline in physical activity at workplaces. Because of this, it is possible to observe several negative effects on functional capacities, as muscle strength, endurance, and flexibility of workers [1]. CWIT is characterized by low and high-intensity interval training (HIIT) of different exercise modalities [59,60], thus promoting beneficial effects on muscle strength and flexibility. Other authors observed that a 12-week moderate intensity exercise training (5 days × week^–1^ with 40 min × session^–1^) improved peak oxygen uptake and BMI in overweight and obese participants [61]. Hence, future studies with other CWIT schemes should examine health-related and body composition outcomes over similar and longer intervention periods. In the present study, it is possible that sex distribution, as well as the lack of nutritional intervention, and the low frequency of CWIT practice (2×/week), may be associated with the absence of changes in body composition parameters. A lower body fatness and an increased muscle mass are most commonly observed in response to more time and days devoted to weekly exercise training regimens [62].

On the other hand, adequate levels of muscle strength are essential to musculoskeletal performance and quality of life. Handgrip strength is an index of the general muscle strength that is easily measured using a hand dynamometer. Handgrip strength has been used for risk stratification to predict individuals’ future health problems [48]. The results from previous studies suggest that more prolonged exercise protocols may be more effective to impact handgrip strength. Previously, a short-term HIIT program (six sessions over two weeks) resulted in negative emotions and exertion in sedentary middle-age men, thus impairing physical and mental well-being [63]. This is contrary to the current study in which the participants exhibited an improved quality of life while experiencing a moderate training load, which highlights the suitability of the current protocol in contrast to other high-intensity exercise modalities.

Moreover, some hypertrophy and sarcomere genesis cannot be disregarded. Growth of filaments and sarcomeres in longitudinal series can influence flexibility development [64], which may be behind the enhanced performance in the sit-and-reach test. In this regard, regular stretching practice at the end of CWIT sessions could have also contributed to this muscle improvement. Another important effect promoted by CWIT was the greater levels of endurance, demonstrated by increased abdominal endurance capacity, and estimated VO_2_max after a 1-mile test on a track. Abdominal fitness test (1-min curl-up test) is performed against body mass load and configures an important index for muscle endurance [45]. The VO_2_max is an important index of functional aerobic capacity. Sedentary individuals have commonly presented lower VO_2_max when compared to active people, and regular exercise training may attenuate these differences [65]. Previously, one study reported that a low-volume HIIT induced significant improvements in VO_2_max, cardiometabolic risk markers and psychological health in physically inactive adults [66], which is in agreement with the current study.

Considering HRV results, total power is considered an estimate of global activity of autonomic nervous system (ANS) [9,11]. HF (0.15 Hz–0.4 Hz) is a marker of cardiac autonomic parasympathetic nervous system activity, while LF (0.04–0.15 Hz) may reflect a combination of both sympathetic and parasympathetic influences [9]. Similarly, a combined aerobic and resistance training promoted significant improvements in HRV parameters in sedentary, hypertensive women [36]. Increased HRV due to exercise training interventions is a potential protection against risk factors and cardiac mortality [67].

Regarding time domain variables, SDNN is a time domain parameter which represents overall variability [9]. On the other hand, RMSSD is the most robust and widely used HRV index and has been reported to be a valid estimate of cardiac vagal modulation [68]. Despite this, RMSSD recordings have revealed large day-to-day variation as a result of different conditions of evaluation [69,70]. Considering the potential effects of evaluating HRV in different positions, it should be noted that the supine position provided more reliable HRV parameters in comparison to other conditions to evaluate HRV in response to training interventions [71,72]. The supine position maximizes the parasympathetic tone, which is important for monitoring in continuous aerobic sports; however, this is not the case of combined demands that rely on anaerobic intermittent bouts (as CWIT) which increase sympathetic activation and vagal withdrawal [27,73]. These differences between different positions with respect to HRV measures [28] may explain the greater changes in HRV parameters observed in the seated vs. supine position. This is an important finding if we consider that our participants mostly work in the seated position.

HRV has been commonly associated with high levels of endurance [45] and several cardiovascular benefits [67], which configure important effects of the CWIT protocol in the present study. Progressive and permanent alterations of HRV in response to extended training periods are an adaptive physiological effect of regular exercise [67,74]. In this aspect, changes in HRV after high-intensity exercise training have generally been associated with inhibition of the sympathetic influences, while aerobic demands are more related to vagal modulations. During exercise training, vagal activity is reduced while sympathetic activity is augmented in response to overload. Afterwards, parasympathetic response is increased after regular exercise training practice in order to normalize heart rate [52]. Like CWIT, interval training is constituted by peaks of high-intensity exercise alternating with periods of low-intensity demands [32,60,62]. As a result, sympathetic and parasympathetic nervous systems must be stimulated in order to provide satisfactory balance and responses to positively adapt to the regular exercise-training.

In this study, CWIT was characterized by a reduced weekly duration and frequency, in comparison to the recommendations of the WHO [38]. This was necessary to increase compliance and to meet the workplace schedule. Despite this, the present intervention was capable of improving the fitness level and quality of life in adults with work-related sedentary behavior. Moreover, it has been confirmed that CWIT maximized fitness benefits in a reduced amount of time [32].

This investigation has some limitations. This is a semi-randomized controlled trial, therefore future randomized controlled trials (RCTs) should confirm our findings in future experiments with more participants of both sexes. Since previous studies have documented substantial impacts of similar protocols on anthropometrical measures [12,62], it may be speculated that the current protocol was suboptimal in terms of frequency of weekly practice. Moreover, it is noteworthy that there was no diet recommendation nor nutritional control during the study development. Therefore, future investigations should consider these factors when looking for improved body composition. In addition, the effects of CWIT on male and female participants were not evaluated separately and this can be also considered an important limitation. Finally, as some fitness parameters such as VO_2_max were estimated from validated formula, future studies should provide objective evaluations of these and other physical and physiological measures (e.g., blood lactate during training sessions) for a better characterization of both acute and chronic adaptations.

## 5. Conclusions

The CWIT used in the current study was associated with improvements in several fitness parameters, including muscle flexibility and aerobic capacity, as well as increases of vitality scores and cardiac autonomic control in adult sedentary workers. Therefore, we may recommend the use of this time-efficient and well tolerated protocol in future interventions with sedentary workers. Examination of different body positions when evaluating changes in HRV after a training intervention, appears to be a relevant aspect to be considered in further studies.

## Figures and Tables

**Table 1:** Protocol for the circuit weight-training workout according to sequence and respective activity performed.

Sequence	Activity			
1.	Data collection (rest) (HR, blood pressure and Borg’s 6–20 RPE scale)			
2.	Warm-up (5 min at 60–70% HRmax in the cycle ergometer)			
3. Station A	Triceps bench dips *	**Sessions 1–8 (1st month)**	**Sessions 9–16 (2nd month)**	**Sessions 17–24 (3rd month)**
		Knees flexed (90°) (3 × 8 reps.; relief intervals: 15 s)	Knees slightly flexed (3 × 10 reps.; relief intervals: 15 s)	Knees completely extended (3 × 13 reps.; relief intervals: 15 s)
	Hip lifts *	Feet on the floor (3 × 8 reps.; relief intervals: 15 s)	Feet on the swiss ball (3 × 10 reps.; relief intervals: 15 s)	Feet on the swiss ball (3 × 13 reps.; relief intervals: 15 s)
	Prone planks *	5 × 10–15 s hold (relief intervals: 10 s)	3 × 20 s hold (relief intervals: 15 s)	3 × 30 s hold (relief intervals: 15 s)
	Cycle ergometer	55 s (60–70% HRmax); 5 s maximal sprint (no load); 30 s maximal sprint (2.5% body mass)	55 s (60–70% HRmax); 5 s maximal sprint (no load); 30 s maximal sprint (4% body mass)	55 s (60–70% HRmax); 5 s maximal sprint (no load); 30 s maximal sprint (5% body mass)
	Data collection (HRmax)			
	Cycle ergometer	3 min easy (no load) intensity cycling (50–70 rpm)		
	Data collection (HR and Borg’s 6–20 RPE scale)			
4. Station B	Standing biceps curl	Load: 1 kg (3 × 8–10 reps.; intervals: 15 s)	Load: 1 kg (3 × 10–13 reps.; relief intervals: 15 s)	Load: 2 kg (3 × 13 reps.; relief intervals: 15 s)
	Dumbbell squats	Load: 0–1 kg (3 × 8–10 reps.; relief intervals: 15 s)	Load: 0–1 kg (3 × 10–13 reps.; intervals: 15 s)	Load: 1–2 kg (3 × 13 reps.; relief intervals: 15 s)
	Pushups *	Knees on the floor (3 × 8–10 reps.; relief intervals: 15 s)	Knees on the floor (3 × 10–13 reps.; relief intervals: 15 s)	Feet on the floor (3 × 8–10 reps.; relief intervals: 15 s)
	Cycle ergometer	55 s (60–70% HRmax); 5 s maximal sprint (no load); 30 s maximal sprint (2.5% body mass); 3 min easy intensity cycling (50–70 rpm)	55 s (60–70% HRmax); 5 s maximal sprint (no load); 30 s maximal sprint (4.0% body mass); 3 min easy intensity cycling (50–70 rpm)	55 s (60–70% HRmax); 5 s maximal sprint (no load); 30 s maximal sprint (5.0% body mass); 3 min easy intensity cycling (50–70 rpm)
	Data collection (HRmax)			
	Cycle ergometer	3 min easy (no load) intensity cycling (50–70 rpm)		
	Data collection (HR and Borg’s 6–20 RPE scale)			
5. Station C	Standing dumbbell lateral raise	Load: 1 kg (3 × 8–10 reps.; relief intervals: 15 s)	Load: 1 kg (3 × 10–13 reps.; relief intervals: 15 s)	Load: 1 kg (3 × 13 reps.; relief intervals: 15 s)
	Dumbbell split squat R leg			Load: 1–2 kg (3 × 13 reps.; relief intervals: 15 s)
	Dumbbell split squat L leg			Load: 1–2 kg (3 × 13 reps.; relief intervals: 15 s)
	Standing dumbbell bent-over row			Load: 2 kg (3 × 13 reps.; relief intervals: 15 s)
	Cycle ergometer	55 s (60–70% HRmax); 5 s maximal sprint (no load); 30 s maximal sprint (2.5% body mass); 3 min easy intensity cycling (50–70 rpm)	55 s (60–70% HRmax); 5 s maximal sprint (no load); 30 s maximal sprint (4.0% body mass); 3 min easy intensity cycling (50–70 rpm)	55 s (60–70% HRmax); 5 s maximal sprint (no load); 30 s maximal sprint (5.0% body mass); 3 min easy intensity cycling (50–70 rpm)
	Data collection (HRmax)			
	Cycle ergometer	3 min easy (no load) intensity cycling (50–70 rpm)		
	Data collection (HR and Borg’s 6–20 RPE scale)			
6.	Cooling down (5 min at an “easy”—no load—intensity in the cycle ergometer)			
7.	Data collection (rest) (blood pressure, HRmax and mean HR)			
8.	Active stretching exercises (9 min; 1 rep × 45 s per position), including muscle in sites of trunk, upper limbs, and lower limbs following this sequence.			

Note. HRmax, maximal heart rate; RPE, rating of perceived exertion; reps., repetitions. * Load: body mass.

**Table 2:** Anthropometrical results according to group and moment.

Variables	Moment	Group		Effect Size (η_p_^2^)
		Control	Experimental	
Body mass (kg)	Initial	78.9 ± 14.7	76.5 ± 15.8	0.005
	Final	78.8 ± 15.0	76.6 ± 15.8	0.005
η_p_^2^		0.001	0.001	
BMI (kg/m²)	Initial	27.7 ± 3.9	27.2 ± 4.4	0.003
	Final	27.7 ± 4.1	27.2 ± 4.4	0.003
η_p_^2^		0.000	0.000	
Arm circumference (cm)	Initial	33.6 ± 3.9	32.8 ± 5.5	0.006
	Final	34.2 ± 3.9	33.4 ± 4.3	0.007
η_p_^2^		0.020	0.056	
Leg circumference (cm)	Initial	39.4 ± 2.0	39.8 ± 3.6	0.003
	Final	39.3 ± 1.9	39.6 ± 3.5	0.002
η_p_^2^		0.005	0.021	
Waist circumference (cm)	Initial	94.5 ± 11.9	88.3 ± 14.3	0.047
	Final	93.4 ± 12.7	87.6 ± 13.5	0.043
η_p_^2^		0.049	0.045	
Hip circumference (cm)	Initial	104.9 ± 6.4	105.0 ± 7.1	0.000
	Final	105.4 ± 6.6	105.5 ± 7.2	0.000
η_p_^2^		0.011	0.019	
Waist-to-hip ratio	Initial	0.90 ± 0.07	0.84 ± 0.10	0.087
	Final	0.88 ± 0.07	0.83 ± 0.09	0.071
η_p_^2^		0.068	0.062	

Note. BMI, body mass index; η_p_^2^, partial eta squared. Values expressed as mean ± standard deviation.

**Table 3:** Physical fitness testing results according to group and moment.

Variables	Moment	Group		Effect Size (η_p_^2^)
		Control	Experimental	
Handgrip strength (kgf)	Initial	35.7 ± 14.8	30.9 ± 11.4	0.033
	Final	37.9 ± 14.8	32.6 ± 11.6 ^#^	0.039
η_p_^2^		0.116	0.163	
Sit and reach test (cm)	Initial	22.9 ± 12.5	22.6 ± 11.4	0.000
	Final	23.2 ± 11.6	25.3 ± 10.1 ^#^	0.009
η_p_^2^		0.004	0.538	
1-min curl-ups test (repetitions)	Initial	15.4 ± 7.9	21.5 ± 8.6	0.107
	Final	15.5 ± 11.1	24.7 ± 8.0 *^,#^	0.191
η_p_^2^		0.000	0.242	
VO_2_max (mL/kg/min)	Initial	33.92 ± 11.47	34.03 ± 5.36	0.000
	Final	33.10 ± 11.93	36.45 ± 6.05 ^#^	0.037
η_p_^2^		0.018	0.275	
Time for completion Rockport Fitness Test (min)	Initial	16.60 ± 1.05	15.90 ± 1.34	0.064
	Final	16.80 ± 1.30	15.37 ± 1.59 *	0.166
η_p_^2^		0.009	0.139	

Note. VO_2_max, estimated maximum oxygen consumption; η_p_^2^, partial eta squared. Values expressed as mean ± standard deviation; * *p* < 0.05 versus control group-final; ^#^ *p* < 0.05 versus experimental group-initial.

**Table 4:** Quality of life results according to group and moment.

Scores	Moment	Groups		Effect Size (η_p_^2^)
		Control	Experimental	
Physical functioning	Initial	69.4 ± 10.1	71.6 ± 11.4	0.009
	Final	66.9 ± 15.8	76.6 ± 8.8 ^#^	0.144
η_p_^2^		0.025	0.196	
Physical role limitations	Initial	87.5 ± 35.3	76.3 ± 32.8	0.024
	Final	84.3 ± 26.5	93.4 ± 14.0 ^#^	0.052
η_p_^2^		0.003	0.194	
Body pain	Initial	77.9 ± 22.2	70.2 ± 18.6	0.033
	Final	68.3 ± 28.4	77.4 ± 18.2	0.039
η_p_^2^		0.053	0.068	
General health perceptions	Initial	71.1 ± 14.2	75.6 ± 14.9	0.020
	Final	71.5 ± 21.4	81.7 ± 13.3 ^#^	0.084
η_p_^2^		0.000	0.220	
Vitality	Initial	64.4 ± 21.4	59.7 ± 17.4	0.014
	Final	59.4 ± 28.6	72.4 ± 13.4 ^#^	0.096
η_p_^2^		0.024	0.276	
Social functioning	Initial	78.1 ± 30.4	87.5 ± 19.9	0.035
	Final	76.5 ± 29.4	90.1 ± 11.5	0.109
η_p_^2^		0.003	0.020	
Emotional role functioning	Initial	87.5 ± 35.3	82.4 ± 30.1	0.006
	Final	75.0 ± 38.8	89.5 ± 22.3	0.057
η_p_^2^		0.047	0.035	
Mental health	Initial	73.5 ± 27.9	73.9 ± 14.7	0.000
	Final	80.5 ± 19.7	83.1 ± 10.6 ^#^	0.008
η_p_^2^		0.054	0.193	

Note. η_p_^2^, partial eta squared. Values expressed as the mean ± standard deviation; ^#^ *p* < 0.05 versus experimental group-initial; two-way repeated measures ANOVA and Bonferroni’s test.

**Table 5:** Analysis of heart rate variability parameters on supine position according to group and moment of evaluation.

Variables		Moment	Groups		Effect Size (η_p_^2^)
			Control	Experimental	
Time domain	SDNN (ms)	Initial	35.70 ± 13.07	31.74 ± 17.50	0.034
		Final	37.04 ± 12.71	41.42 ± 22.85 ^#^	0.000
	η_p_^2^		0.006	0.328	
	RMSSD (ms)	Initial	37.46 ± 19.40	36.51 ± 25.64	0.010
		Final	37.45 ± 14.38	42.31 ± 23.57	0.003
	η_p_^2^		0.002	0.199	
Frequency domain	LF (ms^2^)	Initial	602 ± 334	358 ± 423	0.149
		Final	645 ± 457	841 ± 1044 ^#^	0.004
	η_p_^2^		0.000	0.369	
	HF (ms^2^)	Initial	655 ± 660	629 ± 722	0.015
		Final	585 ± 522	945 ± 1090	0.018
	η_p_^2^		0.018	0.202	
	Total (ms^2^)	Initial	1306 ± 798	1024 ± 1065	0.064
		Final	1294 ± 939	1916 ± 2316 ^#^	0.003
	η_p_^2^		0.006	0.293	
	LF/HF	Initial	1.38 ± 0.74	0.90 ± 0.75	0.086
		Final	1.51 ± 0.83	1.09 ± 0.82	0.057
	η_p_^2^		0.012	0.059	
Non-Linear	SampEn	Initial	1.792 ± 0.137	1.698 ± 0.277	0.032
		Final	1.673 ± 0.212	1.629 ± 0.295	0.006
	η_p_^2^		0.044	0.036	
	DFA Alfa 1	Initial	0.988 ± 0.218	0.836 ± 0.275	0.071
		Final	0.991 ± 0.100	0.948 ± 0.280	0.007
	η_p_^2^		0.000	0.194	

Note. SDNN, standard deviation of normal–normal beats; RMSSD, root-mean-square of successive RR intervals. LF, low frequency; HF, high frequency; LF/HF, ratio between high and low frequencies; η_p_^2^, partial eta squared. Values expressed as the mean ± standard deviation; ^#^ *p* < 0.05 versus experimental group-initial; two-way RM ANOVA and Bonferroni’s test.

**Table 6:** Analysis of heart rate variability at time and frequency domains on sitting position according to group and moment of evaluation.

Variables		Moment	Groups		Effect Size (η_p_^2^)
			Control	Experimental	
Time domain	SDNN (ms)	Initial	38.45 ± 12.70	31.04 ± 12.12	0.086
		Final	36.90 ± 15.53	39.43 ± 17.88 ^#^	0.003
	η_p_^2^		0.018	0.348	
	RMSSD (ms)	Initial	34.56 ± 11.11	29.05 ± 12.75	0.058
		Final	29.04 ± 12.37	34.30 ± 13.73 ^#^	0.032
	η_p_^2^		0.088	0.147	
Frequency domain	LF (ms^2^)	Initial	907 ± 872	486 ± 569	0.136
		Final	875 ± 756	795 ± 789 ^#^	0.012
	η_p_^2^		0.001	0.403	
	HF (ms^2^)	Initial	562 ± 398	434 ± 497	0.064
		Final	460 ± 389	541 ± 397	0.005
	η_p_^2^		0.047	0.120	
	Total (ms^2^)	Initial	1555 ± 1069	995 ± 1066	0.117
		Final	1407 ± 1101	1426 ± 1088 ^#^	0.000
	η_p_^2^		0.016	0.343	
	LF/HF	Initial	1.86 ± 1.36	1.52 ± 1.08	0.018
		Final	2.55 ± 2.22	1.60 ± 1.10	0.082
	η_p_^2^		0.078	0.003	
Non-Linear	SampEn	Initial	1.628 ± 0.175	1.596 ± 0.200	0.092
		Final	1.490 ± 0.225	1.556 ± 0.266	0.020
	η_p_^2^		0.006	0.015	
	DFA Alfa 1	Initial	1.059 ± 0.186	1.007 ± 0.300	0.008
		Final	1.214 ± 0.232	1.083 ± 0.296	0.047
	η_p_^2^		0.126	0.075	

Note. SDNN, standard deviation of normal–normal beats; RMSSD, root-mean-square of successive RR intervals. LF, low frequency; HF, high frequency; LF/HF, ratio between high and low frequencies; η_p_^2^, partial eta squared. Values expressed as the mean ± standard deviation; ^#^ *p* < 0.05 versus experimental group-initial; two-way RM ANOVA and Bonferroni’s test.

**Table 7:** Analysis of heart rate variability in terms of relative variation (%) between supine and sitting positions of evaluation according to group and moment.

Variables		Moment	Groups		Effect Size (EF)
			Control	Experimental	
Time domain	SDNN ^1^	Initial	11.6 (−27.0–50.0)	11.2 (−45.7–100.0)	0.097
		Final	−2.2 (−24.2–24.9)	−1.1 (−16.3–5.9)	0.056
	EF		0.035	0.013	
	RMSSD ^2^	Initial	−0.23 ± 25.39	−5.65 ± 31.02	0.008
		Final	−22.64 ± 10.65 ^#^	−10.34 ± 29.39	0.050
	EF		0.162	0.020	
Frequency domain	LF ^1^	Initial	46.3 (−48.5–189.2)	29.3 (−62.9–423.5)	0.046
		Final	55.8 (−46.1–207.4)	29.4 (−54.4–166.4)	0.066
	EF		0.035	0.966	
	HF ^2^	Initial	19.2 ± 85.7	4.7 ± 66.4	0.009
		Final	25.8 ± 144.4	5.2 ± 74.8	0.010
	EF		0.003	0.000	
	Total ^2^	Initial	31.9 ± 64.0	34.5 ± 82.7	0.000
		Final	30.7 ± 67.4	21.6 ± 67.1	0.004
	EF		0.000	0.021	
	LF/HF ^1^	Initial	59 (−44–196)	50 (−77–1445)	0.148
		Final	56 (−63–548)	49 (−20–1758)	0.005
	EF		0.035	0.013	
Non-Linear	SampEn ^1^	Initial	−9.2 (−20.8–−2.5)	−8.3 (−23.4–72.6)	0.105
		Final	−13.4 (−29.1–35.5)	−2.4 (−22.3–17.2)	0.065
	EF		0.128	0.250	
	DFA Alfa 1 ^1^	Initial	4.3 (−24.4–49.6)	13.0 (−17.1–245.2)	0.169
		Final	15.8 (−1.9–55.1)	10.8 (−4.0–44.2)	0.179
	EF		0.385	0.104	

Note. SDNN, standard deviation of normal–normal beats; RMSSD, root-mean-square of successive RR intervals. LF, low frequency; HF, high frequency; LF/HF, ratio between high and low frequencies; EF, effect size. ^1^ Values expressed as the median and total amplitude; EF expressed as *r* estimated. ^2^ Values expressed as the mean ± standard deviation; EF expressed as partial eta squared (η_p_^2^); ^#^ *p* < 0.05 versus experimental group-initial.

## Data Availability

The data presented in this study are available on reasonable request from the corresponding author. The data are not publicly available due to ethical requirements.

## References

[1] Owen N., Healy G.N., Matthews C.E., Dunstan D.W. (2010). Too much sitting. Exerc. Sport Sci. Rev..

[2] Brown W.J., Bauman A.E., Owen N. (2008). Stand up, Sit down, keep moving: Turning circles in physical activity research?. Br. J. Sports Med..

[3] Cheema B.S., Marshall P.W., Chang D., Colagiuri B., Machliss B. (2011). Effect of an office worksite-based yoga program on heart rate variability: A randomized controlled trial. BMC Public Health.

[4] Després J.-P. (2016). Physical activity, sedentary behaviours, and cardiovascular health: When Will cardiorespiratory fitness become a vital sign?. Can. J. Cardiol..

[5] Blangsted A.K., Søgaard K., Hansen E.A., Hannerz H., Sjøgaard G. (2008). One-year randomized controlled trial with different physical-activity programs to reduce musculoskeletal symptoms in the neck and shoulders among office workers. Scand. J. Work. Environ. Health.

[6] Coury H.J.C.G., Moreira R.F.C., Dias N.B. (2009). Efetividade do exercício físico em ambiente ocupacional para controle da dor cervical, lombar e do ombro: Uma revisão sistemática. Rev. Bras. Fisioter..

[7] Andersen L.L., Saervoll C.A., Mortensen O.S., Poulsen O.M., Hannerz H., Zebis M.K. (2011). Effectiveness of small daily amounts of progressive resistance training for frequent neck/shoulder pain: Randomised controlled trial. Pain.

[8] Sihawong R., Janwantanakul P., Sitthipornvorakul E., Pensri P. (2011). Exercise therapy for office workers with nonspecific neck pain: A systematic review. J. Manip. Physiol..

[9] (1996). Heart Rate Variability: Standards of Measurement, Physiological Interpretation and Clinical Use. Task Force of the European society of cardiology and the North American Society of pacing and electrophysiology. Circulation.

[10] Xhyheri B., Manfrini O., Mazzolini M., Pizzi C., Bugiardini R. (2012). Heart Rate Variability Today. Prog. Cardiovasc. Dis..

[11] Tonello L., Rodrigues F.B., Souza J.W.S., Campbell C.S.G., Leicht A.S., Boullosa D.A. (2014). The role of physical activity and heart rate variability for the control of work related stress. Front. Physiol..

[12] Zaffalon Júnior J.R., Viana A.O., de Melo G.E.L., De Angelis K. (2018). The impact of sedentarism on Heart Rate Variability (HRV) at rest and in response to mental stress in young women. Physiol. Rep..

[13] Garner D.M., Vanderlei F.M., Valenti V.E., Vanderlei L.C.M. (2019). Non-linear regulation of cardiac autonomic modulation in obese youths: Interpolation of ultra-short time series. Cardiol. Young.

[14] Hjortskov N., Rissen D., Blangsted A.K., Fallentin N., Lundberg U., Søgaard K. (2004). The effect of mental stress on heart rate variability and blood pressure during computer work. Eur. J. Appl. Physiol..

[15] Järvelin-Pasanen S., Sinikallio S., Tarvainen M.P. (2018). Heart rate variability and occupational stress—Systematic Review. Ind. Health.

[16] Uusitalo A., Mets T., Martinmäki K., Mauno S., Kinnunen U., Rusko H. (2011). Heart rate variability related to effort at work. Appl. Erg..

[17] Cornelissen V.A., Verheyden B., Aubert A.E., Fagard R.H. (2010). Effects of aerobic training intensity on resting, exercise and post-exercise blood pressure, heart rate and heart-rate variability. J. Hum. Hypertens..

[18] Cornelissen V.A., Goetschalckx K., Verheyden B., Aubert A.E., Arnout J., Persu A., Rademakers F., Fagard R.H. (2011). Effect of endurance training on blood pressure regulation, biomarkers and the heart in subjects at a higher age. Scand. J. Med. Sci. Sports.

[19] Alansare A., Alford K., Lee S., Church T., Jung H. (2018). The effects of high-intensity interval training vs. moderate-intensity continuous training on heart rate variability in physically inactive adults. Int. J. Environ. Res. Public Health.

[20] Besnier F., Labrunée M., Richard L., Faggianelli F., Kerros H., Soukarié L., Bousquet M., Garcia J.-L., Pathak A., Gales C. (2019). Short-term effects of a 3-week interval training program on heart rate variability in chronic heart failure. A randomised controlled trial. Ann. Phys. Rehabil. Med..

[21] Kingsley J.D., Figueroa A. (2016). Acute and training effects of resistance exercise on heart rate variability. Clin. Physiol. Funct. Imaging.

[22] Flatt A.A., Globensky L., Bass E., Sapp B.L., Riemann B.L. (2019). Heart rate variability, neuromuscular and perceptual recovery following resistance training. Sports.

[23] Proper K.I., Staal B.J., Hildebrandt V.H., van der Beek A.J., van Mechelen W. (2002). Effectiveness of physical activity programs at worksites with respect to work-related outcomes. Scand. J. Work. Environ. Health.

[24] Hautala A.J., Karjalainen J., Kiviniemi A.M., Kinnunen H., Mäkikallio T.H., Huikuri H.V., Tulppo M.P. (2010). Physical activity and heart rate variability measured simultaneously during waking hours. Am. J. Physiol. Circ. Physiol..

[25] Falcão J., Sinzato C., Massuda K., Masunaga D., Oliveira Júnior S., Christofoletti G., Carregaro R. (2013). Impactos físicos e mentais de um programa de exercícios terapêuticos direcionado aos servidores de uma instituição pública de mato grosso do sul. Rev. Bras. Ativ. Física Saúde.

[26] Boullosa D., Barros E., del Rosso S., Nakamura F., Leicht A. (2014). Reliability of heart rate measures during walking before and after running maximal efforts. Int. J. Sports Med..

[27] Silva C.C., Bertollo M., Reichert F.F., Boullosa D.A., Nakamura F.Y. (2017). Reliability of heart rate variability in children: Influence of Sex and Body Position During Data Collection. Pediatr. Exerc. Sci..

[28] Rave G., Boullosa D.A., Saeidi A. (2019). Heart rate variability is correlated with perceived physical fitness in elite soccer players heart rate variability is correlated with perceived physical fitness in elite soccer players. J. Hum. Kinet..

[29] De Sousa A.F.M., Medeiros A.R., Benitez-Flores S., Del Rosso S., Stults-Kolehmainen M., Boullosa D.A. (2018). Improvements in attention and cardiac autonomic modulation after a 2-weeks sprint interval training program: A fidelity approach. Front. Physiol..

[30] Barak O.F., Jakowvljevic D.G., Gacesa J.Z.P., Ovcin Z.B., Brodie D.A., Grujic N.G. (2010). Heart Rate Variability Before and After Cycle Exercise in relation to Different Body Positions. J. Sports. Sci. Med..

[31] Medeiros A.R., Leicht A.S., Michael S., Boullosa D. (2020). Weekly vagal modulations and their associations with physical fitness and physical activity. Eur. J. Sport Sci..

[32] Skidmore B.L., Jones M.T., Blegen M., Matthews T.D. (2012). Acute effects of three different circuit weight training protocols on blood lactate, heart rate, and rating of perceived exertion in recreationally active women. J. Sport. Sci. Med..

[33] Paoli A., Paccelli F., Bargossi A.M., Marcolin G., Guzzinati S., Neri M., Bianco A., Palma A. (2010). Effects of three distinct protocols of fitness training on body composition, strength and blood lactate. J. Sports Med. Phys Fit..

[34] Gist N.H., Fedewa M.V., Dishman R.K., Cureton K.J. (2014). Sprint interval training effects on aerobic capacity: A systematic review and meta-analysis. Sports Med..

[35] Abel M.G., Mortara A.J., Pettitt R.W. (2011). Evaluation of circuit-training intensity for firefighters. J. Strength Cond. Res..

[36] Masroor S., Bhati P., Verma S., Khan M., Hussain M.E. (2018). Heart rate variability following combined aerobic and resistance training in sedentary hypertensive women: A randomised control trial. Indian Heart J..

[37] Arslan S.S., Alemdaroglu I., Karaduman A.A., Yilmaz Ö.T. (2019). The effects of physical activity on sleep quality, job satisfaction, and quality of life in office workers. Work.

[38] WHO (2020). Guidelines on Physical Activity and Sedentary Behaviour.

[39] Shephard R.J. (1988). PAR-Q, Canadian home fitness test and exercise screening alternatives. Sport. Med..

[40] Schulz K.F., Altman D.G., Moher D. (2010). CONSORT 2010 statement: Updated guidelines for reporting parallel group randomised trials. J. Clin. Epidemiol..

[41] Cherkas A., Abrahamovych O., Golota S., Nersesyan A., Pichler C., Serhiyenko V., Knasmüller S., Zarkovic N., Eckl P. (2015). The correlations of glycated hemoglobin and carbohydrate metabolism parameters with heart rate variability in apparently healthy sedentary young male subjects. Redox Biol..

[42] Borg G.A. (1982). Psychophysical bases of perceived exertion. Med. Sci. Sports Exerc..

[43] Scherr J., Wolfarth B., Christle J.W., Pressler A., Wagenpfeil S., Halle M. (2013). Associations between Borg’s rating of perceived exertion and physiological measures of exercise intensity. Eur. J. Appl. Physiol..

[44] Tingelstad H.C., Theoret D., Spicovck M., Haman F. (2016). Explaining performance on military tasks in the Canadian armed forces: The importance of morphological and physical fitness characteristics. Mil. Med..

[45] Lam C.L.K., Gandek B., Ren X.S., Chan M.S. (1998). Tests of scaling assumptions and construct validity of the Chinese (HK) version of the sf-36 health survey. J. Clin. Epidemiol..

[46] Kline G.M., Porcari J.P., Hintermeister R., Freedson P.S., Ward A., McCarron R.F., Ross J., Rippe J.M. (1987). Estimation of VO2max from a one-mile track walk, gender, age, and body weight. Med. Sci. Sports Exerc..

[47] Faulkner R.A., Sprigings E.J., McQuarrie A., Bell R.D. (1989). A partial curl-up protocol for adults based on an analysis of two procedures. Can. J. Sport Sci..

[48] Vanderburgh P.M., Mahar M.T., Chou C.H. (1995). Allometric scaling of grip strength by body mass in college-age men and women. Res. Q. Exerc. Sport.

[49] Wells K.F., Dillon E.K. (1952). The sit and reach—A test of back and leg flexibility. Res. Q. Am. Assoc. Heal. Phys. Educ. Recreat..

[50] Giles D., Draper N., Neil W. (2016). Validity of the polar V800 heart rate monitor to measure RR intervals at rest. Eur. J. Appl. Physiol..

[51] Quintana D.S., Heathers J.A.J. (2014). Considerations in the assessment of heart rate variability in biobehavioral research. Front. Psychol..

[52] Lima-Borges D.S., Martinez P.F., Vanderlei L.C.M., Barbosa F.S.S., Oliveira-Junior S.A. (2018). Autonomic modulations of heart rate variability are associated with sports injury incidence in sprint swimmers. Phys. Sportsmed..

[53] Richardson J.T.E. (2011). Eta squared and partial eta squared as measures of effect size in educational research. Edu. Res. Rev..

[54] Pollock M.L., Gaesser G.A., Butcher J.D., Després J.-P., Dishman R.K., Franklin B.A., Garber C.E. (1998). ACSM position stand: The recommended quantity and quality of exercise for developing and maintaining cardiorespiratory and muscular fitness, and flexibility in healthy adults. Med. Sci. Sport. Exerc..

[55] Gotshalk L.A., Berger R.A., Kraemer W.J. (2004). Cardiovascular responses to a high-volume continuous circuit resistance training protocol. J. Strength Cond. Res..

[56] Kay G.N., Ashar M.S., Bubien R.S., Dailey S.M. (1995). Relationship between heart rate and oxygen kinetics during constant workload exercise. Pacing Clin. Electrophysiol..

[57] Gearhart R.E., Goss F.L., Lagally K.M., Jakicic J.M., Gallagher J., Robertson R.J. (2001). Standardized scaling procedures for rating perceived exertion during resistance exercise. J. Strength Cond. Res..

[58] Focht B.C. (2007). Perceived exertion and training load during self-selected and imposed-intensity resistance exercise in untrained women. J. Strength Cond. Res..

[59] Giannaki C.D., Aphamis G., Sakkis P., Hadjicharalambous M. (2016). Eight weeks of a combination of high intensity interval training and conventional training reduce visceral adiposity and improve physical fitness: A group-based intervention. J. Sports Med. Phys. Fit..

[60] Monks L., Seo M.-W., Kim H.-B., Jung H.C., Song J.K. (2017). High-intensity interval training and athletic performance in taekwondo athletes. J. Sports Med. Phys. Fit..

[61] Kozey Keadle S., Lyden K., Staudenmayer J., Hickey A., Viskochil R., Braun B., Freedson P.S. (2014). The independent and combined effects of exercise training and reducing sedentary behavior on cardiometabolic risk factors. Appl. Physiol. Nutr. Metab..

[62] Burrup R., Tucker L.A., Cheminant J.D.L.E., Bailey B.W. (2018). Strength training and body composition in middle-age women. J. Sports Med. Phys. Fit..

[63] Saanijoki T., Nummenmaa L., Eskelinen J.-J., Savolainen A.M., Vahlberg T., Kalliokoski K.K., Hannukainen J.C. (2015). Affective responses to repeated sessions of high-intensity interval training. Med. Sci. Sport. Exerc..

[64] O’Sullivan K., McAuliffe S., DeBurca N. (2012). The effects of eccentric training on lower limb flexibility: A systematic review. Br. J. Sports Med..

[65] Herdy A.H., Caixeta A. (2016). Brazilian cardiorespiratory fitness classification based on maximum oxygen consumption. Arq. Bras. Cardiol..

[66] Shepherd S.O., Wilson O.J., Taylor A.S., Thøgersen-Ntoumani C., Adlan A.M., Wagenmakers A.J.M., Shaw C.S. (2015). Low-volume high-intensity interval training in a gym setting improves cardio-metabolic and psychological health. PLoS ONE.

[67] Vrachimis A., Hadjicharalambous M., Tyler C. (2016). The effect of circuit training on resting heart rate variability, cardiovascular disease risk factors and physical fitness in healthy untrained adults. Health.

[68] Goldberger J.J., Challapalli S., Tung R., Parker M.A., Kadish A.H. (2001). Relationship of Heart rate variability to parasympathetic effect. Circulation.

[69] Sandercock G.R.H., Bromley P.D., Brodie D.A. (2005). The reliability of short-term measurements of heart rate variability. Int. J. Cardiol..

[70] Tonello L., Reichert F.F., Oliveira-Silva I., Del Rosso S., Leicht A.S., Boullosa D.A. (2016). Correlates of heart rate measures with incidental physical activity and cardiorespiratory fitness in overweight female workers. Front. Physiol..

[71] Plews D.J., Laursen P.B., Le Meur Y., Hausswirth C., Kilding A.E., Buchheit M. (2014). Monitoring training with heart-rate variability: How much compliance is needed for valid assessment?. Int. J. Sports Physiol. Perform..

[72] Flatt A.A., Esco M.R. (2015). Smartphone-derived heart-rate variability and training load in a women’s soccer team. Int. J. Sports Physiol. Perform..

[73] Abad C., Kobal R., Kitamura K., Gil S., Pereira L., Loturco I., Nakamura F. (2017). Heart rate variability in elite sprinters: Effects of gender and body position. Clin. Physiol. Funct. Imaging.

[74] Vanderlei L.C.M., Pastre C.M., Hoshi R.A., de Carvalho T.D., de Godoy M.F. (2009). Noções básicas de variabilidade da frequência cardíaca e sua aplicabilidade clínica. Rev. Bras. Cir. Cardiovasc..

